# The ligamentum teres—its increasing importance

**DOI:** 10.1093/jhps/hnu003

**Published:** 2014-07-30

**Authors:** John M. O'Donnell, Michael Pritchard, Antonio Porthos Salas, Parminder J. Singh

**Affiliations:** 1. 21 Erin Street, Richmond, Victoria 3121, Australia; 2. Hip Arthroscopy Australia, 21Erin St Richmond, Australia; 3. Hip Arthroscopy Australia, 30 Cascade Rd South Hobart, Hobart, Australia

**Keywords:** ligamentum teres, hip arthroscopy

## Abstract

The ligamentum teres (LT) has attracted much greater interest over recent years due to the increased use of hip arthroscopy. There have been advancements in our understanding of the LT’s biomechanical function and its role in hip and groin pain. Our ability to suspect LT tears by clinical examination and imaging has improved. Publications by many authors concerning LT tear treatment and outcomes continue to increase. This manuscript is a review of the function, mechanism of injury, clinical assessment, imaging, arthroscopic assessment, treatment, outcomes, reconstruction, and unusual conditions of the LT.

## INTRODUCTION

The ligamentum capitis femoris or ligamentum teres (LT) was known in ancient times. Hegetor, an Alexandrian medical writer (130 BC), recognized the round ligament of the hip joint [1]. In April, 1874 Professor W.S. Savory presented a paper to the Cambridge Philosophical Society regarding his theory on the function of the LT of the hip joint. This theory was immediately challenged by Professor Humphry, and still there remains no universal agreement regarding the function of this ligament. However, hip arthroscopy has allowed direct visualization of the LT in its intact state, and this has led to heightened interest in both its function and clinical importance. Since the comprehensive review publication of the LT of the adult hip by Bardakos and Villar [2] in 2009, there have been a number of advances in our understanding of this ligament. This manuscript is a review of the function, mechanism of injury, clinical assessment, imaging, arthroscopic assessment, treatment, outcomes, reconstruction and unusual conditions of the LT.

## FUNCTIONAL ROLE OF THE LT

In 1874 Savory [3] suggested that the LT acted as a suspensory ligament to diminish pressure between the head of the femur and the roof of the acetabulum in animals with an erect posture. The function of the ligament, since this publication, has remained somewhat controversial; however, recent work has added to our understanding of the role of LT, which continues to be gradually better defined.

Anatomically, the LT predominantly arises from the transverse acetabular ligament along the inferior margin of the acetabulum. The LT is attached by two bands that are located along the ischial and pubic margins of the acetabular notch [4]. In its acetabular aspect, the ligamentum is flat and pyramidal. The LT transitions into a round and ovoid shape when it attaches to the fovea capitis. The overall length of the LT averages 30–35 mm [5].

Mechanical testing of the LT has been reported to demonstrate some similarity in structure and strength to the anterior cruciate ligament of the knee. The strength of the porcine LT has been tested and was found to be similar to the human ACL, with an average load to failure of 882 N [6].

**Figure 1.**
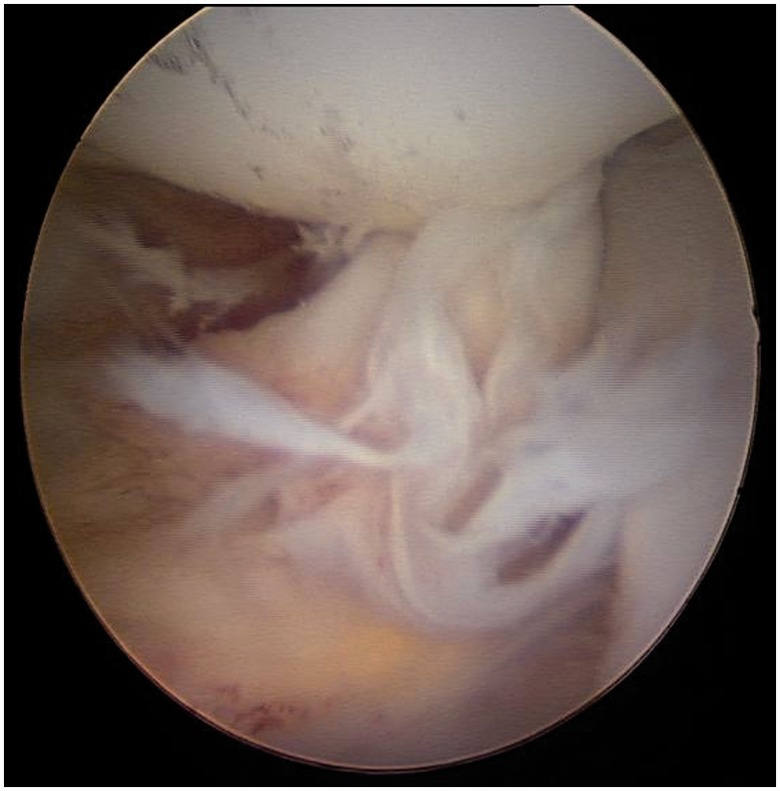
Normal LT demonstrating two-bundle structure.

The LT has also been shown to contain free nerve endings thought to indicate both a proprioceptive and nociceptive role for the ligament [7, 8]. Using immunohistochemistry, Haversath *et al*. [9] in 2013 confirmed the presence of nociceptive and proprioceptive innervation of the LT (as well as the labrum and capsule) and found that these nerves were concentrated in the centre of the ligament. The ligamentum is considered particularly strong, even at birth, as a result of its collagenous formation, it is composed of collagen type I, III, IV. Gray and Villar [10] suggested that by virtue of the topography of the fovea the ligamentum is tightest in adduction, flexion and external rotation of the hip. As this is the position in which the hip joint is least stable, a mechanical role of the ligamentum was proposed in contributing to hip stability.

Earlier cadaveric studies using ligament sectioning techniques reported by Demange *et al.* [5] found that abduction–adduction, but particularly adduction, of the hip was increased after sectioning the ligament in seven cadavers. Although the difference was significant, the change was small (mean 3.5°) and of uncertain importance or clinical relevance. In addition, in animal models, when the LT is severed, the rate of hip dislocation has been shown to increase [6]. In humans, however, a torn LT has not been proven to increase the risk for dislocation.

More recently, Martin *et al*. [11] in 2012 used string models to assess the excursion of the LT during hip movements. The model found the string to have its greatest excursion when the hip was externally rotated in flexion (ER/FLEX) and internally rotated in extension (IR/EXT). The authors concluded that the LT may contribute to hip stability, particularly when the hip is in ER/FLEX and IR/EXT. In addition, the authors studied 20 patients found at arthroscopy to have complete LT ruptures and identified an osseous abnormality that appeared to correlate with symptomatic instability. They further concluded that individuals with osseous risk factors for instability, including inferior acetabular insufficiency, may have instability with squatting (ER/FLEX) and crossing one leg behind the other (IR/EXT). The authors have argued that the role of the LT in hip stability may in fact become more important when other stabilizers were deficient, such as when there was diminished bony stability (anteroinferior acetabular deficiency) or ligamentous stability (generalized ligamentous laxity).

In a separate article, Kivlan *et al*. [12] used human cadavers to demonstrate that when the human hip moves into flexion-abduction, the LT moves into a position that provides anterior and inferior stabilization of the hip. ‘The ligamentous endpoint determined for the LT resembled the position of the hip joint when performing a squat. From the endpoint position the LT formed a “sling-like” structure that supported the femoral head inferiorly and prevented anterior/inferior subluxation of the hip joint’.

As well as its potential role in hip stability, Gray and Villar [10] suggested a possible role for the LT in synovial fluid circulation, the so-called windshield wiper effect. However, there is a paucity of evidence supporting this theory.

## MECHANISMS OF INJURY

To date, the proposed mechanisms of injury to the LT comprise traumatic injury, iatrogenic injury sustained during open surgical dislocation and repetitive ‘microtrauma’ often associated with hyperlaxity. More recent work by Domb *et al.* [13] has reviewed the relationship of LT tears with acetabular morphology and its significance. Manner *et al*.’s [14] work has emphasized the significance of Femoro-acetabular Impingement (FAI). Others have presented proposed mechanisms including impingement against bony prominences or articular cartilage, but this work has not yet been published.

Most reports of traumatic cases contain case reports or very small case series [15–17]. For example, major trauma leading to hip dislocation has been reported as a cause of LT tear. In contrast, others have reported relatively minor trauma, such as shopping with a supermarket trolley as a cause [18]. In the absence of bony morphological abnormality, extreme range of movement of the hip may occur during activities performed, for example, by gymnasts and martial artists. These movements may lead to repetitive microtrauma to the LT. LT tears are also more frequently found in patients with hyperlaxity or ligamentous insufficiency, such as Ehlers-Danlos disease [19].

Haviv and O’Donnell [20] and Amenabar and O’Donnell [21] reported treatment of isolated LT tears by debridement alone. The authors found an incidence of recurrent LT tears, which they postulated may be due to persisting microinstability. They found that further treatment with anterior capsular tightening appeared to reduce this risk of recurrent tears.

Bony morphological abnormality, of the acetabulum, femur or both bones, may also predispose to tears of the LT. Arthroscopy undertaken on patients with acetabular dysplasia has demonstrated an increased incidence of LT tears as well as labral tears [22]. Engaging cam lesions have also been suggested to cause a degree of posterior hip subluxation, leading to increased strain and tear of the LT [14].

In a study looking at risk factors and LT tears, Domb *et al*. [23] suggested that LT tears were less common in patients below 30 years of age and in those with acetabular retroversion and increased prominence of the ischial spine. These authors also found a positive link with increased risk of tearing and decreased lateral coverage index (LCI). This is defined as the centre edge angle minus acetabular inclination. Patients with a low LCI were 1.74 times more likely to have tears than those with high LCI hips.

In a group of patients with degenerative arthritis, attrition tears of the LT may occur as a result of abrasion against degenerate osteophytes around the edge of the acetabular fossa. In the absence of degenerative change, O’Donnell and Economopoulos (International Society for Hip Arthroscopy Annual Scientific Meeting, Boston, 2012) have presented a further mechanism of LT tears, where mid-substance tears of the posterior part of the LT were associated with impingement against a prominent bony ridge on the posteroinferior portion of the wall of the acetabular fossa.

## CLINICAL ASSESSMENT

LT tears have been extremely difficult to diagnose pre-operatively. Baber *et al*. [24] in 1999 found 4% of cases at arthroscopy had LT tears, but none were diagnosed pre-operatively. Although most authors have stated that there are no definitive features in the history to diagnose a LT tear, some features may be suggestive. A history of a twisting injury or fall on a flexed knee is more common, but an episode involving hyperabduction should also raise clinical suspicion. The patient may complain of groin pain related to activity [25], sometimes radiating to the medial thigh [26], and may describe mechanical symptoms, such as painful clicking, locking or giving way. Others have suggested that LT tears may commonly be associated with radiation of pain to the buttock [20].

Disabling symptoms are not always present, especially in athletes [26]. Martin *et al.* [11] reported a series of arthroscopically proven LT ruptures (all associated with FAI) and demonstrated symptoms of instability in five out of nine. All five had feelings of instability when squatting, and four of those five described a feeling of instability when crossing their involved leg behind their uninvolved leg when standing.

The LT has been sacrificed by those surgeons performing open dislocation surgery for FAI for many years. Generally, it appears to have been accepted that this has no ill effect. However, Phillips *et al*. [27] described a series of 161 patients who they surveyed after open hip dislocation surgery, during which the LT was sectioned. Thirty-five percent of these patients described popping and locking, and 24% had subjective feelings of the hip giving way. These were described by the authors as minor instability feelings.

Examination findings associated with LT tear have been described as non-specific signs of hip irritability such as pain provocation with flexion, adduction and internal rotation movement, and with the log-roll test.

Clinical examination may show a reduced and painful range of movement of the hip joint, either in extension or in combined flexion and internal rotation [25]. Tests indicative of intra-articular pathology may be positive in the presence of LT tears, but are non-specific. These include the log-roll test, resisted straight-leg raise and McCarthy’s tests, where, with both hips flexed, the affected hip is passively brought to extension, first in internal and then in external rotation [28].

Botser *et al*. [29], however, demonstrated no statistically significant difference in examination findings, including range of movement, in patients with and without LT tears.

There has, however, been no accepted clinical test for LT tear. There has even been disagreement about the likelihood of positive non-specific signs, as demonstrated by the earlier papers.

O’Donnell *et al*. [30] have described a new clinical test that aims to detect LT pathology specifically known as the LT test. The examined hip is flexed to approximately 70° and abducted 30°, and the knee flexed to 90°. Then the hip is rotated internally and externally to its full extent. Pain provocation represents a positive test. The authors suggest that in this position there should be no bony impingement and that the LT will be selectively tightened.

**Figure 2.**
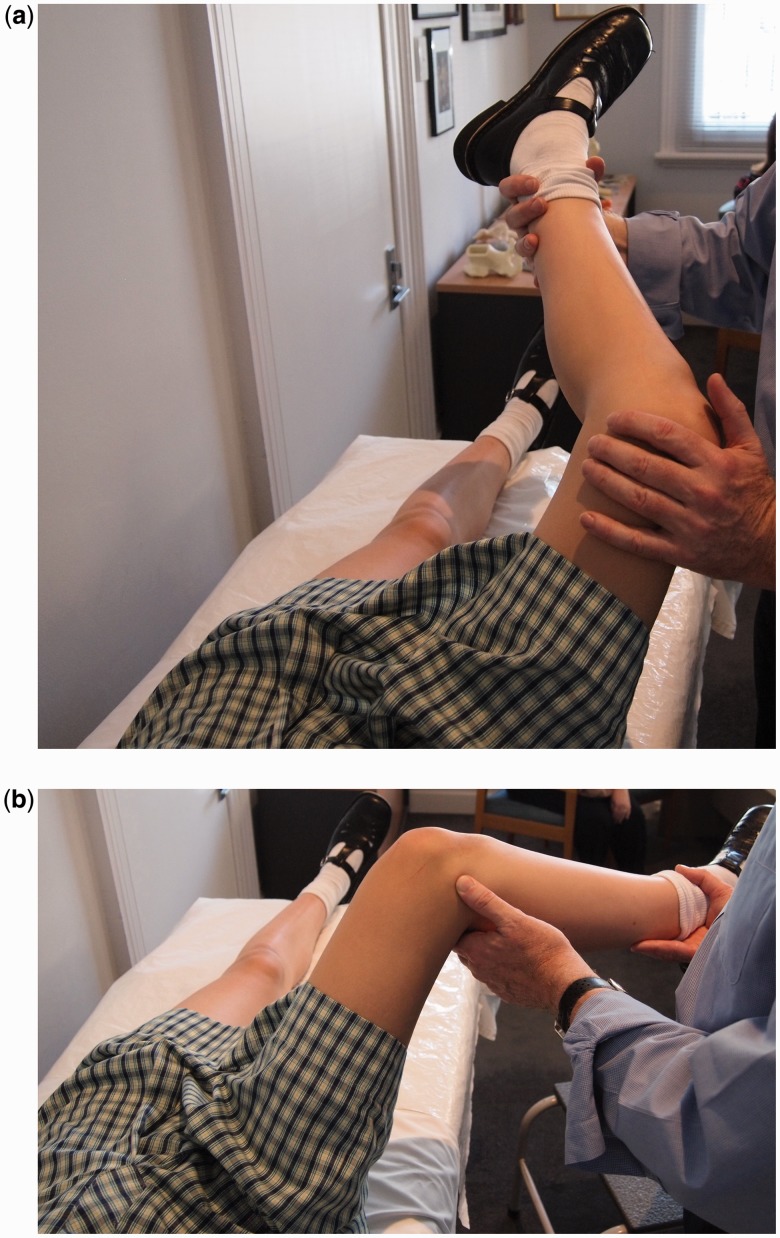
LT test. The hip is flexed to 70° and abducted 30°. The knee is flexed to 90°. (a) and (b) have been reversed relative to the pictures (a) is external rotation of the hip and (b) is internal rotation of the hip.

This would appear to be consistent with the work of Martin *et al*. [11] described previously. The LT test results were compared with arthroscopic findings in 75 patients, specifically looking for LT tears, both partial and complete, and synovitis around the LT. A positive predictive value of 84% was found and a negative predictive value of 91%. A positive test was also seen in a patient with a large pincer lesion and unstable labral tear, but no LT tear.

Lampert [31], in the German literature, briefly described a test for LT tear, the loaded rotation test of the hip in which the patient is standing, with weight on the tested leg. With the leg straight, the tested hip is rotated internally. A 78% positive result was claimed.

**Figure 3.**
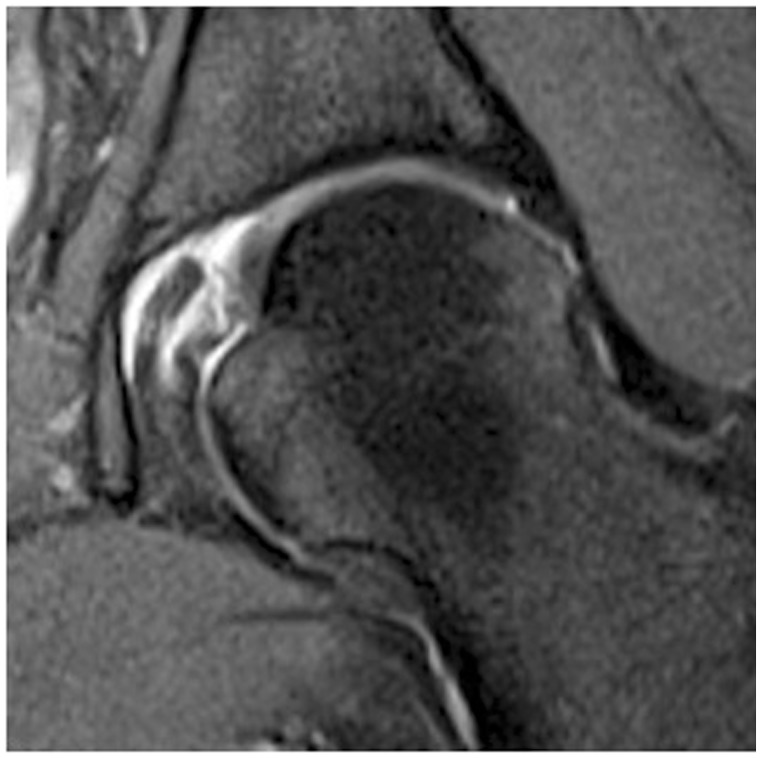
LT partial thickness tear-coronal oblique Proton Density MRI showing linear signal and intrinsic signal changes to foveal insertion of LT.

## IMAGING

There have been many changes in imaging of the hip aiming to improve the accuracy of diagnosis. Along with improvements in magnetic resonance imaging (MRI) and magnetic resonance arthrography (MRA) with increasingly powerful magnets, new techniques have been introduced to change the study of articular cartilage, such as dGEMRIC and T2 mapping. However, imaging of the LT has remained problematic and unreliable.

Imaging of the LT has not proved reliable in predicting pathology. Byrd and Jones [25] reported a pre-operative diagnosis of LT tears in only 2/41, which included 20 MRIs and 7 MRAs.

Botser *et al*. [29] report tears of the LT prevalence in hip arthroscopy using two different classification systems. The authors reported a study of 558 hip arthroscopies and found an incidence of LT tears of 51%. Pre-operative MR (mixed MRI and MRA) was reported as diagnosing nine cases of LT tear, and only five of these were confirmed at arthroscopy. Athroscopic interest in the ligament as a source of pathology and pain has heightened interest and awareness. Improvements in MRI sequences and protocols may increase reliability, and more recent papers appear to show some improvement in the ability to diagnose LT tears pre-operatively.

Several authors have commented on the difficulties of diagnosing tears of the LT, particularly partial thickness tears. Blankenbaker *et al*. [32] compared MRA findings and hip arthroscopy findings in 116 patients, and concluded that the intact and partially torn LT can have similar findings on MRA, making the diagnosis of partial LT tears difficult. High signal intensity within the substance of the fibres and irregularity suggest partial thickness tear; however, the authors reported that further research was warranted. Cerezal *et al*. [33] suggested that MRA and Computerised axial tomographic arthrography (CTA) are superior to MR and CT for demonstrating LT tears.

Devitt *et al*. [34] in 2014 reported that, using a 3-T MRI scanner and specific sequencing, the sensitivity of MRI to diagnose tears of the LT was much higher than was previously reported. Specifically, the sensitivity of MRI to diagnose partial tears was 91%, and it had a 78% sensitivity in identifying hypertrophy of the LT. MRI showed moderate sensitivity and specificity of 50% and 34%, respectively, in identifying any pathologic process of the LT. MRI is capable of detecting partial tears of the LT with high sensitivity (91%) and positive predictive value (67%). It remains for others to reproduce these results.

There has also been some interest in the use of distraction with MRA. In 1983, Vegter and Van Den Broek [35] described a technique using manual traction applied to the leg whilst taking a radiograph of the hip. This technique has been refined by Lopis *et al*. [36] who used skin traction with a 6 kg weight hanging over the end of the platform to provide distraction and MRA. They described the technique to improve the visualization of the articular cartilage of the femoral head and acetabulum. It may also help to improve the diagnosis of LT pathology.

## ARTHROSCOPIC ASSESSMENT

Bardakos and Villar [2] suggested that an adequate assessment of the LT required direct arthroscopic visualisation, direct vision of the LT under stress, internal and external rotation, and also careful probing. Lesions of the LT can arise from the origin, insertion or through the substance of the ligament. A number of descriptions of the lesions to the LT have been described: partial or complete traumatic tears, degenerative tears, avulsion fractures of the ligament at its insertion into the fovea capitis femoris and a congenital absence of the ligament.

**Figure 4.**
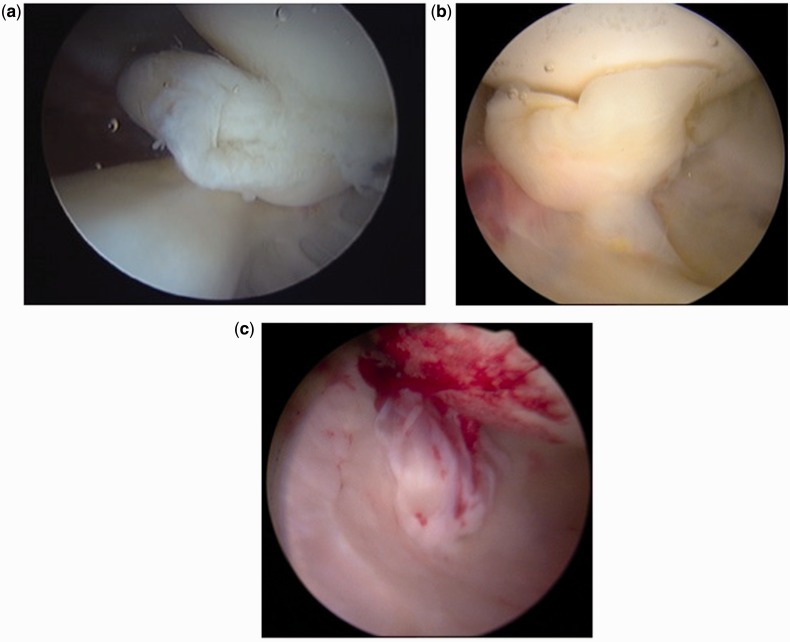
(a) Type 1, Complete (b) Type 2 Partial Thickness and (c) Type 3 Degenerate tears.

Gray and Villar [10] introduced a classification of LT tears in 1997 (see Figure 4), consisting of Grade 1 (complete), Grade 2 (partial) and Grade 3 (degenerate), Botser *et al.* [29] have suggested refining this classification with the addition of partial 2a <50%, and partial 2b >50% tears of the LT.

Despite this recommended way of assessing the LT, there remains an enormous variation in the reported incidence of LT tears seen at arthroscopy, ranging from 8% to 51%. The reasons for this variation have not been determined, but it seems likely that the reasons for the higher rates described may include increasing awareness of partial thickness tears, more thorough assessment of the LT and perhaps also a lower threshold for applying the term partial thickness tears.

Few authors have looked in detail at the mechanism of injury beyond trauma and degeneration. Dynamic assessment of the LT during hip arthroscopy allows the surgeon to make a further evaluation of other possible mechanisms of injury of the LT. For example, the authors of this review have observed a number of LT that impinge against a projection of the posterior wall of the acetabular fossa during internal rotation. This impingement has been associated with localized synovitis and mid-substance fraying of the LT. This pathology is different from most of the partial thickness tears which we have observed that occurred at the femoral attachment of the LT, and are most easily seen with the hip in external rotation. 

## TREATMENT

Recommended treatment for LT tears has been simply debridement with either mechanical shavers or radiofrequency (RF) probes [2]. Where a specific cause of the tear can be identified, or suggested, that cause should also be treated. This may include capsule tightening in the presence of instability [21] or periacetabular osteotomy in the presence of hip dysplasia [22].

In the presence of a large cam lesion, abutment of the acetabulum and proximal femur can lead to subtle subluxation of the hip joint posteriorly. This in turn can produce tightening and or a tear in LT. Resection of the cam lesion in this type of injury to the LT would be recommended to avoid recurrent injury.

## OUTCOME OF TREATMENT

In 2004 Byrd and Jones [25] reported a case series study of 23 patients with LT injuries. A 100% follow-up rate was seen at an average of 29.2 months (range, 12–60 months). Patients included 14 women and 9 men, with an average age of 28.3 years (range, 15–53 years). Fifteen patients sustained violent trauma (7 motor vehicle accidents, 3 falls from a height, 3 football injuries, 1 snow skiing injury, 1 ice hockey injury), including 6 dislocations. The remaining eight patients sustained a twisting injury. Duration of symptoms before surgery averaged 28.5 months (range, 0.5–144 months).

Arthroscopic findings comprised ligament injury was an isolated finding in 8 cases and associated pathology was noted in 15 cases (9 labral tears, 5 loose bodies, 5 chondral damage). Overall, the average pre-operative modified Harris Hip Score (MHHS) score of 47 improved to 90 postoperatively, which was statistically significant (*P* < 0.001). Ninety-six percent (22 patients) showed 20-point improvement. No statistically significant difference was seen between patients with major trauma or twisting injuries, complete or partial ruptures, isolated lesions or concomitant pathology. According to this study, Byrd argued that regardless of the cause and of the often associated pathology, hip problems in which disruption of the LT is implicated as a contributing source may respond well to arthroscopic intervention. 

In 2010 Haviv and O’Donnell [20] reported a case series of 29 patients with isolated LT tear. Patients with acetabular dysplasia and all other intra-articular pathologies were excluded. These exclusion criteria were in contrast to the study reported by Byrd *et al*. [25], which included all associated pathologies within their cohort. Clinical results were measured pre-operatively and post-operatively with the MHHS and Non-Arthritic Hip Score (NAHS). The mean age was 25 years (SD ± 11) with a mean follow-up time of 2.5 years (SD ± 1.5). Review of the demographic data revealed that those with partial LT ruptures occurred mostly in young women who typically were involved in activities such as gymnastics, calisthenics and dancing.

At the last follow-up, the mean MHHS improved from 70 to 86 [mean difference = 16 (95% CI 4–27)] and the mean NAHS improved from 64 to 86 mean difference = 22 (95% CI 10–33)]. Five patients had a second arthroscopic debridement due to symptomatic recurrent tears. Recurrent tears were treated by re-debridement and capsular tightening. The authors reported that whenever a tear of the LT is detected, it is customary to treat the torn segment and surrounding synovitis by debridement or shrinkage.

The authors concluded that arthroscopic debridement alone of the isolated LT rupture has a short-term beneficial result in more than 80% of cases. The recurrence rate of a LT tear was 17% using this technique.

In a follow-up article, Amenabar and O’Donnell [21] treated a series of 27 similar patients with isolated partial thickness LT tears using a combination of RF debridement and additional anterior capsulorraphy using either thermal (RF) or suture capsulorraphy. Both the RF and suture patients had similar outcomes. In this group of patients, there were no tear recurrences over an average 32-month follow-up period. They suggested that in the first group of patients recurrent LT tears may have been due to persisting instability. In the second group, the tightening of the capsule led to 10′–15′ diminished range of external rotation, and may have led to improved stability, and the observed diminished risk of tear recurrence.

In 2009, Philippon *et al*. [37] reported a retrospective case series chart review of the arthroscopic findings following traumatic hip dislocation in 14 professional athletes. Eighty-five percent of this cohort were posterior dislocations and 15% were anterior dislocations. The mean time from dislocation to reduction was 3.56 h. The mean time from dislocation to surgery was 125 days (range, 0–556 days). The average age at the time of arthroscopy was 30.5 years (range, 16–46 years). All patients had labral tears. All patients had chondral defects. Eleven patients had partial or complete tears of the LT. The authors concluded that their results showed that traumatic dislocation was accompanied by a variety of intra-articular hip joint pathologies, the most common being labral, chondral, intra-articular loose fragments and disruption of the LT. This study was a retrospective case series study with all of the limitations that entails. Furthermore, the authors did not know whether the dislocation was the sole cause of the arthroscopic findings for each patient. The authors argue that they may well have been pre-existing underlying issues including femoroacetabular and chondrolabral dysfunction and have no way of confirming or disproving this finding.

## LT RECONSTUCTION

Our improved understanding of the role of the LT and its structure has led to an interest in reconstructing the ligament in selected cases with symptomatic instability and an absent LT. LT reconstruction was first described [38] by Philippon *et al.* [39] in 2012 but remains a rarely performed operation, with uncertain indications, minimal outcome data and no agreement regarding technique. LT reconstruction techniques have been described by five authors. The indication for this surgery has been persisting instability associated with known LT rupture. Contra-indications have been significant acetabular dysplasia and degenerative arthritis. The papers predominantly have been descriptions of techniques, with few outcome results. All used interference screw fixation within a femoral tunnel, but varied in their choice of graft and acetabular fixation method. Philippon *et al*. [39] initially described a technique using an iliotibial band (ITB) graft, fixed with an anchor to the acetabular fossa. 

Simpson *et al*. [38] used a synthetic graft fixed with an endobutton within the pelvis. This technique allowed fixation of the acetabular end of the graft within a short bony tunnel in the acetabular floor, and they felt provided more secure fixation. They also included in their article extremely important cadaveric work regarding the risk of drilling through the acetabular fossa floor to place the endobutton, and concluded that, with due care, this can be done safely with low risk to the intrapelvic vessels.

Amenabar and O’Donnell [40] described a semitendinosus tendon graft fixed with two anchors to the acetabular floor. These authors suggested that as the LT had many structural features in common with ACL, it was appropriate to use a graft that had been shown to be successful in ACL reconstruction in the knee. Lindner *et al*. [41] used semitendinosus similarly, but with a retrobutton to fix the acetabular end of the graft.

Mei-Dan and McConkey [42] described the use of all suture anchors on the acetabular side of the reconstruction. They suggested that this was a safer option as these smaller and softer anchors were less likely to cause intra-pelvic or intra-articular complications.

The ideal position of the leg for graft tensioning is also uncertain. These authors suggested a range of extension from 10° to neutral, and a range of external rotation from 20° to 60°. The ideal tensioning position and method of tensioning remain to be determined.

Only Philippon *et al*. [39] has so far published results of this procedure. He reported four patients with an ITB graft. One with acetabular dysplasia (centre edge angle 14′) and degenerative change failed and required total hip replacement. The other three reported improvement from 1 to 3 years, although the MHHSs were incomplete.

## UNUSUAL LT CONDITIONS

Of some interest, Bardakos and Villar [2] reported that Villar had seen four cases of an LT in anatomical continuity after severe injury, including dislocation. We have also seen three cases of an apparently intact LT after known rupture and total excision performed previously by us. This would suggest that the LT does have some propensity to heal and reform after injury. The factors that may lead to this occurrence are unknown.

There have also been two reports of benign tumours of the LT. In 2009 Singh *et al.* [43] reported the first case in the literature of a giant cell tumour (GCT) of the LT. A GCT of the LT was readily visualized and removed arthroscopically with complete resolution of symptoms. A second soft tissue tumour of the LT was reported by McLawhorn *et al*. [44] In this case, a fibromyxoid pseudotumour of LT led to significant bony erosion, which required osteochondral grafting, in addition to removal of the tumour.

 Finally, Ebraheim *et al*. [45] reported post-traumatic calcification of LT mimicking a loose body.

## CONCLUSION

Although our knowledge of the precise role and function of the LT remains incomplete, our understanding of this ligament is increasing. It is now known to be a potential source of pain when damaged, and new, innovative treatments have been described to treat it. In addition to further basic science work and improved imaging, there is also a need for further outcome studies of treatment and, particularly, longer-term outcome results.
